# The effect of the subclinical small ruminant lentivirus infection of female goats on the growth of kids

**DOI:** 10.1371/journal.pone.0230617

**Published:** 2020-03-24

**Authors:** Tomasz Nalbert, Michał Czopowicz, Olga Szaluś-Jordanow, Lucjan Witkowski, Agata Moroz, Marcin Mickiewicz, Iwona Markowska-Daniel, Danuta Słoniewska, Emilia Bagnicka, Jarosław Kaba

**Affiliations:** 1 Division of Veterinary Epidemiology and Economics, Institute of Veterinary Medicine, Warsaw University of Life Sciences, Warsaw, Poland; 2 Division of Infectious Diseases, Department of Small Animal Diseases with Clinic, Institute of Veterinary Medicine, Warsaw University of Life Sciences, Warsaw, Poland; 3 Institute of Genetics and Animal Breeding, Polish Academy of Sciences, Magdalenka, Poland; University of Minnesota, UNITED STATES

## Abstract

A longitudinal observational study was carried out to evaluate the influence of prenatal exposure to small ruminant lentivirus(SRLV)-infected does on the body weight (BWT) of young kids. The study was carried out in years 2001–2017 in the research dairy goat herd. Goats in the herd were regularly serologically tested and individuals showing clinical signs of caprine arthritis-encephalitis (CAE) were promptly culled. As a result all goats enrolled in the study were asymptomatic. Moreover, kids were weaned immediately after birth, fed on bovine colostrum and kept in strict separation from mothers to prevent SRLV lactogenic transmission. Kids were weighed immediately after birth, and then 1–3 times within the first 3 months of life. In total 620 goat kids were weighed at least once, excluding weighing at birth, providing 992 BWT records. The mixed linear model including four variables fitted as random effects (doe, kid, the year of kid’s birth and the exact age of a kid at weighing) and four potential confounders fitted as fixed effects (parity, kid’s sex, litter size and birth body weight) was developed and showed that BWT was not significantly associated with SRLV serological status of a doe, regardless of the time for which does had been infected before the delivery of the kid (p = 0.242). This study provides strong evidence that kids born to SRLV-infected does grow equally well as kids from uninfected does, provided that the lactogenic viral transmission is prevented by maintaining strict separation between the offspring and mothers. This observation is important for choosing the most optimal strategy of CAE control in a goat herd.

## Introduction

Small ruminant lentiviruses (SRLV) are a broad group of retroviruses able to infect small ruminants. Even though they cause two clinically different diseases–maedi-visna in sheep and caprine arthritis-encephalitis (CAE) in goats–SRLV are capable of crossing the interspecies barrier and spreading from sheep to goats and vice versa [1,2]. Both diseases may take severe clinical course and be eventually fatal, however clinical signs develop after several-year long incubation period and only in part of infected animals [3]. Since much more animals are affected by a subclinical infection than by an apparent disease, the knowledge of influence of subclinical SRLV infection on productivity of goats is vital for the development of economically-balanced CAE control programs.

Eradication of SRLV from a goat herd is extremely laborious and expensive. Therefore, test-and-cull control programs are successfully completed mostly in these regions where governmental assistance is available [4,5]. Otherwise, farmers interested in CAE control restrict themselves to programs based on early weaning and mother-offspring separation along with elimination of adult goats presenting clinical signs, assuming that costs of the test-and-cull eradication program would quickly outweigh costs of the presence of subclinical infection in the herd. To evaluate if such an opinion is justified the knowledge of influence of subclinical infection on goat productivity is crucial.

While several studies have investigated the link between SRLV infection and milk yield or milk quality [6–8], so far only one has described its influence on the growth of kids [9]. It revealed that growth rate of kids from seropositive does was significantly lower both before and after weaning at the age of 3 months. On the other hand, this problem has been extensively studied in ewes affected by maedi-visna disease and results appear to be contradictory. Even though most of studies did not show any link between SRLV infection and decreased lamb productivity [10–13], some revealed its detrimental effect on body weight and growth rate of lambs at weaning [14–16]. In all the aforementioned studies kids and lambs used to be left with mothers for 2–3 months after birth. During this time they were constantly exposed to SRLV in their dams’ milk. As the lactogenic route plays a main role in SRLV transmission [17], these studies made indeed no distinction between the influence of mother’s infection and the influence of early infection of a lambs or kids on their growth rate.

The theoretical rationale for hypothesizing the link between the mother’s infection and kid’s growth is based on some observations made over last decades in pregnant women infected with human immunodeficiency virus (HIV) which is a close relative of SRLV. The growth of HIV-exposed but uninfected children seems to be impaired, mainly in the first 12–24 months of their life [18–21]. Pathogenesis of this phenomenon is not clear with a chronic immune activation in response to prolonged prenatal HIV exposure [22] or an exposure to antiretroviral drugs *in utero* and postnatally during breastfeeding [23] being the most likely culprits. One prominent difference between pathogenesis of lentiviral infections in humans and goats is that the epitheliochorial placenta of goats protects fetuses against direct intrauterine infection with SRLV. On the other hand, a chronic immune activation seems to take place in infected goats [24,25]. Whether it affects their fetuses remains unknown.

Therefore, we decided to carry out an observational longitudinal study to evaluate the influence of prenatal exposure of kids to SRLV-infected does on the body weight of these kids up to the age of 3 months.

## Materials and methods

### Goats

This observational study was carried out in years 2001–2017 in the Polish research dairy goat herd counting each year approximately 50–60 does of the Polish White Improved and Polish Fawn Improved breed. The goats were housed in a large and tall brick loose barn, with a very efficient natural ventilation system, regularly changed thick straw bedding, and free access to a few fenced grassy backyards. The goats were fed according to the INRA system [26] mainly with the mixed ration based on corn silage, haylage, mixed concentrates and whole-grain oat. Fresh water and mineral licks were constantly accessible for all goats. The goats were milked mechanically twice a day in a parallel milking parlor. For the entire study period the same small group of workers cared for adult goats and kids. Does were mated yearly in September-October, for the first time when they reached body weight of at least 30 kg and the age of at least 6 months.

Type of rearing of kids was the same for the entire study period and involved mother-offspring separation. Kids were weaned immediately after birth, before they consumed colostrum, and fed on bovine colostrum for 3–4 days. Then, they were switched to the milk replacer based on cow or sheep milk (Dolmilk Ovi, Dolfos, Poland and SprayFo, Trouw Nutrition, Poland) which was given 3 times a day for the first month, then tapered off over next weeks and discontinued at the age of 3 months. In the second week of life hay, and starting from the third week of life mixed concentrates based on oat were gradually introduced. Kids were weighed before the first consumption of bovine colostrum and then within the first 3 months of life in 3 time points: 1) between the age of 21 and 35 days which was referred to as weighing at the age of 1 month; 2) between the age of 50 and 64 days which was referred to as weighing at the age of 2 months; 3) between the age of 77 and 91 days which was referred to as weighing at the age of 3 months. A portable electronic animal plate weighing scale with weighing precision of ±0.05 kg (model SD75L, Ohaus Corporation, Parsippany, NJ, USA) was used and the body weight of each kid (BWT) was recorded in kg with one decimal place. BWT records from kids which died during the observation period were discarded as potentially affected by pathological conditions other than SRLV infection.

### SRLV infection

SRLV infection was detected in this herd by serological and virological methods over 20 years ago [27]. At the beginning of the 21^st^ century the voluntary control program based on regular serological monitoring, weaning of kids immediately after birth and separation of offspring from their dams was launched and has been continued for the entire period of this study. This control program is described in detail elsewhere [28]. All goats in the herd were tested serologically first at the age of 4–6 months and then once or twice a year (in spring and autumn) using the commercial ELISA kit: ELISA Checkit CAEV/MVV (Dr. Bommeli AG, Bern, Switzerland) in years 2001–2007; Pourquier ELISA Maedi-Visna/CAEV Serum Verification (Institut Pourquier, Montpellier, France) in years 2008–2012; ID Screen MVV-CAEV Indirect Screening test (ID.vet Innovative Diagnostics, Grabels, France) in years 2013–2017. All assays were performed according to manufacturers’ manuals on two microplate readers–the ICN Flow Titertek Multiscan Plus Mk11 (Labsystems, Espoo, Finland) in years 2001–2012, and the Epoch Microplate Spectrophotometer (BioTek, USA) in years 2013–2017. Their results were interpreted according the cut-off points recommended by the manufacturers. At these cut-off points sensitivity and specificity of the three assays was as follows: 98.6% and 99.3% for Checkit ELISA [29], 98.8% and 97.2% for Pourquier ELISA [30], and 91.7% and 98.9% for ID Screen ELISA [31]. The change of ELISA kits during the study period resulted from their availability on the market and the financial policy of the laboratory responsible for serological testing. Adult goats were culled as soon as any clinical signs of CAE (mainly arthritis resulting in markedly and consistently impaired mobility) had become apparent. As a result all infected goats enrolled in the study were asymptomatic. SRLV present in the herd has lately been classified into a novel subtype A17 of the genetic group A (maedi-visna virus-like) [32]. The blood collection was approved by the 3^rd^ Local Ethical Committee in Warsaw (Approvals No. 44/2009, 31/2013).

### Statistical analysis

Numerical variables were presented as the arithmetic mean and standard deviation (±SD) or the median and interquartile range (IQR) depending on the normality of their distribution. Range was shown in all cases. Univariable analysis of the relationship between kid’s body weight and potential confounders such as kid’s sex or litter size was performed using the analysis of covariance with the exact age of a kid at weighing as a covariate, followed by the Tukey’s HSD or Dunnett’s post-hoc test. For each category of potential confounders crude arithmetic means (±SD) and arithmetic means adjusted by the exact age of a kid at weighing were presented with the 95% confidence interval (CI 95%). Categorical variables were presented as counts and percentages in categories and compared using the Pearson’s chi-square test.

The mixed linear model (MLM) was developed to assess the relationship between BWT and SRLV infection in a doe. Following variables were forced into MLM and fitted as random effects: i) a variable “kid” nested in a variable “doe” to control for the dependence of measurements obtained from a single kid at different time intervals as well as from the different number of kids born by the same doe; ii) variable “the year of kid’s birth” to control for the influence of possibly different environmental and management conditions in different breeding seasons; iii) the numerical variable “exact age of a kid at weighing” to control for variation in the exact day of life on which BWT was measured.

Then, four potential confounders were included in MLM as fixed effects: “kid’s sex” included as a category of males (X_males_) with females as a reference category; “birth body weight of a kid” (X_BW_) included as a numerical variable; “litter size” included as two ordinal categories–twins (X_tw_) and triplets/quadruplets (X_tr/Q_) with singletons as a reference category; and “parity” (X_parity_) included as a numerical variable. These variables were tested according to the backward stepwise elimination procedure and the variable with the p-value >0.05 and the lowest F statistics was dropped first.

The main explanatory variable describing SRLV serological status of a doe was entered into MLM as a categorical variable classified into five ordinal categories corresponding to the number of years for which a doe had been seropositive before a kid was born: seropositive for 1 year (X_SRLV-1year_), seropositive for 2 years (X_SRLV-2years_), seropositive for 3 years (X_SRLV-3years_), seropositive for 4 years (X_SRLV-4years_), and seropositive for 5 or more years (X_SRLV-5years_). Seronegative does constituted a reference category. Initial MLM had a following form:

$${\text{Y}}_{\text{BWT}}={\text{B}}_{0}+\left({\text{B}}_{\text{males}}\times {\text{X}}_{\text{males}}+{\text{B}}_{\text{tw}}\times {\text{X}}_{\text{tw}}+{\text{B}}_{\text{tr}/\text{Q}}\times {\text{X}}_{\text{tr}/\text{Q}}+{\text{B}}_{\text{BW}}\times {\text{X}}_{\text{BW}}+{\text{B}}_{\text{parity}}\times {\text{X}}_{\text{parity}}\right)+{\text{B}}_{\text{SRLV}-1\text{year}}\times {\text{X}}_{\text{SRLV}-1\text{year}}+\ldots +{\text{B}}_{\text{SRLV}-n\text{years}}\times {\text{X}}_{\text{SRLV}-n\text{years}}+K\left(D\right)+W+A+\varepsilon$$

B_0_ was an intercept and B with a relevant subscript stood for the coefficient of regression of a given explanatory variable, *K*(*D*) was the random effect of a variable “kid” nested in a variable “doe”, *W* was the random effect of the year in which a kid was born, and *A* was the random effect of the exact age (in days) at which a given kid was weighed. Epsilon letter (*ε*) signified the residual component (error). Parentheses indicated confounders which were retained in or removed from the model on the basis of the backward stepwise elimination procedure. Assumptions of the model were verified.

All statistical tests were two-tailed and the significance level (α) was set at 0.05. Univariable statistical analysis was performed in TIBCO Statistica 13.3.0 (TIBCO Software Inc., Palo Alto, CA, USA). MLM was developed in IBM SPSS Statistics 24 (IBM Corporation, Armonk, NY, USA).

## Results

One hundred thirty four individual female goats were included in the analysis. Of them 66 (49.3%) remained seronegative for the entire observation period, and the another 68 (50.7%) seroconverted. Records from 1 to 7 parities with the median of 2 parities (IQR from 1 to 4 parities) were collected.

Six hundred twenty goat kids were weighed at least once within the first 3 months of life and their records were included in the study. Of them 307 (49.5%) were born to seronegative goats. Remaining 313 kids (50.5%) were born to goats seropositive for the various period of time. Detailed characteristics of kids born to does from different categories are presented in Table 1. In total 992 BWT were recorded– 542 (54.7%) from 1-month-old kids, 301 (30.3%) from 2-month-old kids, and 149 (15.0%) from 3-month-old kids.

**Table 1:** General characteristics of the study population of goat kids.

Serological class of a mother	n (%) of kids born	n of males born (% of all kids born to mothers of a particular serological class)	Litter size ^a^ (% of all kids born to mothers of a particular serological class)			n (%) of kids whose body weight was recorded			n (%) of records of body weight collected	n of records of body weight collected at the age of: (% of all records collected from kids born to mothers of a particular serological class)		
			S	Tw	Tr or Q	once	twice	three times		1 month	2 months	3 months
Seronegative	307 (49.5)	152 (49.5)	62 (20.2)	195 (63.5)	50 (16.3)	160 (52.1)	118 (38.4)	29 (9.5)	483 (48.7)	260 (53.8)	151 (31.3)	72 (14.9)
Seropositive from 1 year	131 (21.1)	74 (56.5)	14 (10.7)	90 (68.7)	27 (20.6)	66 (50.4)	53 (40.4)	12 (9.2)	208 (21.0)	119 (57.2)	57 (27.4)	32 (15.4)
Seropositive from 2 years	52 (8.4)	26 (50.0)	4 (7.7)	26 (50.0)	22 (42.3)	25 (48.1)	15 (28.8)	12 (23.1)	90 (9.1)	48 (53.3)	34 (37.8)	8 (8.9)
Seropositive from 3 years	54 (8.7)	29 (53.7)	6 (11.1)	29 (53.7)	19 (35.2)	25 (46.3)	22 (40.7)	7 (13.0)	73 (7.4)	38 (52.1)	19 (26.0)	16 (21.9)
Seropositive from 4 years	32 (5.2)	13 (40.6)	4 (12.5)	18 (56.2)	10 (31.3)	20 (62.5)	9 (28.1)	3 (9.4)	91 (9.2)	52 (57.1)	23 (25.3)	16 (17.6)
Seropositive from 5 years or more	44 (7.1)	28 (63.6)	2 (4.6)	20 (45.5)	22 (50.0)	18 (40.9)	23 (52.3)	3 (6.8)	47 (4.7)	25 (53.2)	17 (36.2)	5 (10.6)
Total	620 (100)	322 (51.9)	92 (14.8)	378 (61.0)	150 (24.2)	314 (50.6)	240 (38.7)	66 (10.7)	992 (100)	542 (54.6)	301 (30.4)	149 (15.0)
p-value of chi-square test		0.305	<0.001^b^			0.106				-		

^a^ S–singletons, Tw–twins, Tr–triplets, Q–quadruplets.

^b^ positively correlated with parity which is in turn positively correlated with the years from seroconversion

Only 66 kids (10.7%) were weighed at all three time points. Most of kids– 314 (50.6%)–were weighed at only one time point: 247 kids (78.7% of 314 kids) at the age of 1 month, 60 kids (19.1% of 314 kids) at the age of two months, and 7 kids (2.2% of 314 kids) at the age of 3 months. The remaining 240 kids (38.7%) were weighed at two time points: 164 kids (68.3% of 240 kids) at the age of 1 and 2 months, 65 kids (27.1% of 240 kids) at the age of 1 and 3 months, and 11 kids (4.6% of 240 kids) at the age of 2 and 3 months. All the raw data are provided in the S1 Table.

In the univariable analysis BWT proved to be higher in males than in females at all three time points, and higher in singletons than in twins and triplets/quadruplets but only in 1-month-old kids (Table 2). Neither at the age of 1, 2 nor 3 months did BWT differ significantly between serological categories of does (Table 3).

**Table 2:** General characteristics of body weight of kids (BWT) in three different time points—Observed and adjusted by the exact age at which a kid was weighed.

	Birth		1 month-old			2 month-old			3 month-old		
	n (%)	Observed mean ± SD (range)	n (%)	Observed mean ± SD (range)	Adjusted mean (CI 95%) for the fixed mean age of 27 days	n (%)	Mean ± SD (range)	Adjusted mean (CI 95%) for the fixed mean age of 58 days	n (%)	Mean ± SD (range)	Adjusted mean (CI 95%) for the fixed mean age of 82 days
Overall	620	3.74±0.70 (1.8–6.0)	542	7.72±1.83 (3.4–13.8)	-	301	11.79±2.37 (5.4–17.8)	-	149	15.39±2.83 (9.3–30.0)	-
Litter size											
Singletons (S)	92 (14.8)	4.24±0.68 (2.7–5.9)	79 (14.6)	8.69±1.65 (4.8–12.6)	8.62 (8.25–8.99)	32 (10.6)	12.73±2.28 (6.0–17.3)	12.73 (11.97–13.50)	31 (20.8)	16.59±3.73 (10.5–30.0)	16.72 (15.73–17.72)
Twins (Tw)	378 (61.0)	3.76±0.66 (1.8–6.0)	327 (60.3)	7.69±1.76 (3.4–13.8)	7.71 (7.53–7.89)	183 (60.8)	11.73±2.45 (5.4–17.8)	11.83 (11.51–12.16)	86 (57.7)	15.08±2.52 (9.3–22.3)	15.12 (14.53–15.72)
Triplets and quadruplets (Tr/Q)	150 (24.2)	3.38±0.60 (1.8–5.0)	136 (25.1)	7.22±1.89 (4.2–12.2)	7.27 (6.98–7.55)	86 (28.6)	11.55±2.16 (8.0–16.7)	11.45 (10.98–11.91)	32 (21.5)	15.05±2.37 (11.0–20.0)	14.87 (13.88–15.86)
p-value of ANOVA^a^ / ANCOVA^b^ with Tukey’s HSD post-hoc test:	-	<0.001^a^	-	-	<0.001^b^	-	-	0.019^b^	-	-	0.014^b^
S vs. Tw		<0.001			<0.001			0.166			0.079
S vs. Tr/Q		<0.001			<0.001			0.079			0.072
Tw vs Tr/Q		<0.001			0.055			0.840			0.999
Sex											
Males	322 (51.9)	3.93±0.69 (1.8–6.0)	284 (52.4)	8.16±1.96 (4.2–13.8)	8.18 (7.99–8.38)	154 (51.2)	12.20±2.53 (5.4–17.8)	12.14 (11.79–12.49)	79 (53.0)	15.75±3.15 (10.0–30.0)	15.83 (15.20–16.47)
Females	298 (48.1)	3.53±0.66 (1.8–5.7)	258 (47.6)	7.23±1.55 (3.4–12.2)	7.21 (7.00–7.41)	147 (48.8)	11.36±2.12 (6.0–17.3)	11.42 (11.06–11.77)	70 (47.0)	14.97±2.38 (9.3–21.5)	14.89 (14.22–15.56)
p-value of Student’s t-test^a^ or ANCOVA^b^	-	<0.001^a^	-	-	<0.001^b^	-	-	0.005^b^	-	-	0.046^b^

**Table 3:** Body weight of kids (BWT) born to does of different serological status in three different time points observed and adjusted by the exact age at which a kid was weighed.

	Birth		1 month-old			2 month-old			3 month-old		
	n (%)	Observed mean ± SD (range)	n (%)	Observed mean ± SD (range)	Adjusted mean (CI 95%) for the fixed mean age of 27 days	n (%)	Mean ± SD (range)	Adjusted mean (CI 95%) for the fixed mean age of 58 days	n (%)	Mean ± SD (range)	Adjusted mean (CI 95%) for the fixed mean age of 82 days
Seronegative	307 (49.5)	3.83±0.69 (1.8–6.0)	260 (48.0)	7.81±1.83 (3.4–13.8)	7.75 (7.54–7.97)	151 (50.2)	11.65±2.52 (5.4–17.8)	11.70 (11.35–12.05)	72 (48.3)	15.53±3.28 (9.3–30.0)	15.57 (14.92–16.22)
Seropositive from 1 year	131 (21.1)	3.66±0.65 (2.5–5.3)	119 (22.0)	7.88±1.71 (4.5–12.2)	7.84 (7.53–8.15)	57 (18.9)	11.96±2.25 (7.6–17.8)	11.99 (11.41–12.56)	32 (21.5)	15.62±1.91 (11.7–19.6)	15.68 (14.71–16.65)
Seropositive from 2 years	52 (8.4)	3.59±0.71 (2.3–5.2)	52 (9.6)	7.71±1.79 (4.7–12.6)	7.55 (7.07–8.02)	23 (7.6)	12.70±1.95 (9.5–16)	12.54 (11.64–13.45)	16 (10.7)	15.15±2.83 (9.5–21.0)	15.08 (13.71–16.45)
Seropositive from 3 years	54 (8.7)	3.68±0.69 (2.1–5.4)	48 (8.9)	7.16±2.03 (4.4–12.1)	7.31 (6.82–7.81)	34 (11.3)	10.88±2.08 (8.2–16.5)	11.04 (10.30–11.79)	8 (5.4)	13.05±1.54 (11.0–14.7)	13.11 (11.18–15.04)
Seropositive from 4 years	32 (5.2)	3.71±0.75 (2.0–5.2)	25 (4.6)	8.01±2.00 (4.7–11.7)	8.00 (7.32–8.68)	17 (5.6)	12.32±2.53 (8.6–16.1)	12.30 (11.25–13.36)	5 (3.4)	12.9±1.34 (11.0–14.0)	12.88 (10.44–15.31)
Seropositive from 5 years or more	44 (7.1)	3.58±0.78 (1.8–5.1)	38 (7.0)	7.12±1.77 (4.4–10.8)	7.24 (6.69–7.8)	19 (6.3)	12.41±1.80 (9.5–16.3)	12.35 (11.36–13.35)	16 (10.7)	16.46±2.09 (13.3–20.0)	16.40 (15.03–17.77)
Total	620		542			301			149		
p-value of ANOVA^a^ / ANCOVA^b^ with Dunnett’s post-hoc test: seronegative vs:		0.031			0.215			0.099			0.032
Seropositive from 1 year		0.329									0.999
Seropositive from 2 years		0.488									0.991
Seropositive from 3 years		0.874									0.081
Seropositive from 4 years		0.978									0.184
Seropositive from ≥5 years		0.525									0.699

Litter size and parity were excluded from MLM on the basis of backward stepwise elimination procedure (S2 Table). Controlling for the birth body weight of a kid (F_1,641_ = 193.47, p<0.001), kid’s sex (F_1,601_ = 16.25, p<0.001) and random effects, BWT in first 3 months of life did not prove to be significantly associated with SRLV serological status of a doe (F_5,610_ = 1.35, p = 0.242). Detailed results of the model are presented in Table 4.

**Table 4:** Mixed linear model investigating the influence of SRLV infection of a doe on the body weight of an individual kid within the first three months of life (BWT).

Variable	Estimate of the model ^a^	Parameter statistics	p-value
Intercept	6.48 ± 0.66 (5.16, 7.79)	-	-
Variables fitted as fixed effects			
Kid’s sex			
female ^b^	0	-	-
male	0.51 ± 0.13 (0.26, 0.75)	4.03	<0.001*
Birth body weight (BW)	1.32 ± 0.10 (1.13, 1.50)	13.91	<0.001*
Serological status of a doe:			
seronegative ^b^	0	-	-
seropositive for 1 year	0.30 ± 0.16 (-0.02, 0.61)	1.87	0.062
seropositive for 2 years	0.32 ± 0.23 (-0.14, 0.78)	1.38	0.168
seropositive for 3 years	-0.10 ± 0.22 (-0.54, 0.33)	-0.46	0.643
seropositive for 4 years	0.28 ± 0.29 (-0.29, 0.85)	0.96	0.339
seropositive for ≥5 years	0.35 ± 0.25 (-0.14, 0.83)	1.41	0.160
Variables fitted as random effects			
Kid nested in Doe	1.06 ± 0.13 (0.83, 1.36)	8.01	<0.001*
Year of kid’s birth	0.66 ± 0.30 (0.27, 1.62)	2.19	0.028
Exact age of a kid at weighing	11.30 ± 2.47 (7.36, 17.34)	4.57	<0.001*
Residue	1.48 ± 0.11 (1.28, 1.70)	13.90	<0.001*

^a^ regression coefficient (±SE, with CI 95%) for variables fitted as fixed effects and variance (±SE, with CI 95%) for variables fitted as random effects

^b^ reference category

* significant at α = 0.05

## Discussion

In this study we showed that subclinical SRLV infection of a doe did not affect the body weight of kids in the first 3 months of life provided that the offspring were separated from their dam. The study was carried out on the substantial number of goat kids, with a strict control of the most important confounders, and of the time for which a doe had been infected before a kid was born. We believe that these facts make our conclusions highly trustworthy. Along with our previous observations regarding the lack of influence of SRLV infection of a doe on the litter size and the birth body weight of kids [33], this study provides important knowledge of the impact of CAE on goat farming. Our results indicate that using infected mothers for breeding is very unlikely to have negative impact on the development of kids born to them provided that kids are not exposed to lactogenic infection. This observation substantiates the usefulness of control programs based on mother-offspring separation.

It must be stressed that our study did not investigate an influence of early SRLV infection of kids on their growth. Indeed, the vast majority of kids in our study were free from the virus because they had been kept clear of contaminated colostrum or milk. Therefore, the epidemiological situation we investigated was more like the situation of HIV-exposed during pregnancy but uninfected children, than like the situation of lambs in meat or wool sheep flocks enrolled in the cited studies [10–16], since the lambs were constantly exposed to lactogenic transmission of SRLV from their infected dams. We suppose that this fact may explain why some studies have revealed quite strong link between SRLV infection of a dam and impaired growth of the offspring [9,14,15,16]. It seems to be simply because the offspring were exposed to the infection with SRLV from the very beginning of their life. It has been well-evidenced that early infection with lentiviruses e.g. HIV may compromise children’s growth [34].

Three technical aspects of our study need to be clarified. First, the use of serological tests provides only a very rough knowledge of the time for which goats have been infected as the seroconversion after lentiviral infections may be considerably delayed [35]. Therefore, ordinal categories indicating the number of years for which a goat had been seropositive before a kid was born should be treated with caution, rather as an a proxy of the progressive course of CAE, than as an exact indicator of the time necessary for SRLV infection to become sufficiently severe to compromise kids’ growth. Secondly, the age limit of 3 months which we established in our study was dictated mostly by practical circumstances. In this herd goat kids used to be sold to other herds when they turned 3 months and no further follow-up was possible. Most of studies so far carried out in sheep have also covered analogical [16] or even shorter [10,15] period of time. And thirdly, also the reason for which not all the kids were weighed at the same age was purely practical. Except for measuring the birth body weight, weighing of kids was a routine procedure performed by goat caretakers at their convenience–on a given day of week rather than on the exact day of kids’ life. We believe that including the exact age of a kid at weighing allowed us to control for this variability.

The fact that this study was conducted in a single herd could have some influence on the results. Firstly, for the entire 17 years this goat herd has had very good environmental conditions, well-organized and highly-automatized milking parlor, balanced nourishing and last but not least hard-working caretakers truly fond of animals they looked after. It is possible that such a positive surrounding mitigated to some extent the negative impact of CAE on animals. Nevertheless, we suppose that poor management and environmental conditions would have rather increased the proportion of clinically diseased goats in a herd and in this manner negatively affected productivity, than worsened early consequences of prenatal exposure to SRLV-infected mothers. Secondly, since goats in this herd were infected with SRLV genotype A, our study investigated only the consequences of the infection of does with this particular viral genotype. However, no differences in the virulence of genotype A and B have so far been shown [1], while the other genotype pathogenic for goats (E) has even proven to be much less virulent [36]. Therefore, it appears to be very unlikely that infection with other than A genotype could lead to any more detrimental consequences.

Two potential confounders proved significantly linked to the growth of kids in the first 3 months of life–birth body weight and kid’s sex. In our previous study [37] the birth body weight turned out to contribute to the growth of kids during the first four months of life, which is even longer than the time period covered by this study. In many former studies male kids have been born heavier [33,38] and have remained heavier for the entire life [39,40]. On the other hand, even though singletons are usually heavier than kids from multiple litters at birth [33,41] and at weaning [40], in our study kids from litters counting more than one individual caught up with singletons not later than at the age of two months. As a result litter size turned out to be an insignificant confounder in the MLM. We suppose that this may have been resulted from the high quality of nutrition and good environmental conditions in this herd.

Concluding, this study provides strong evidence that kids born to SRLV-infected does grow equally well as kids from uninfected does, provided that the lactogenic viral transmission is prevented by maintaining strict separation between the offspring and mothers. This observation is important for choosing the most optimal strategy of CAE control in a goat herd.

## Supporting information

S1 Table Raw data used in the study. (XLSX)

S2 Table Mixed linear model (MLM) at subsequent steps of backward stepwise elimination procedure. (DOCX)
