# Supplementary material for: The effect of the subclinical small ruminant lentivirus infection of female goats on the growth of kids
Source: PLoS One. 2020 Mar 24;15(3):e0230617. doi: 10.1371/journal.pone.0230617 (PMC7092990; doi:10.1371/journal.pone.0230617)
Supplement: S2 Table — (DOCX) [file pone.0230617.s002.docx]

Supplementary file 2. Mixed linear model (MLM) at subsequent steps of backward stepwise elimination procedure

| Variable | MLM – 1^st^ step  (initial model) | MLM – 2^nd^ step | MLM – 3^rd^ step  (final model) |
| --- | --- | --- | --- |
| Kid’s sex | F_1,597_ = 15.45, p<0.001 | F_1,601_ = 16.08, p<0.001 | F_1,601_=16.25, p<0.001 |
| Birth body weight of a kid (BW) | F_1,631_ = 161.00, p<0.001 | F_1,640_ = 191.54, p<0.001 | F_1,641_=193.47, p<0.001 |
| Litter size | F_2,630_ = 0.89, p=0.412^a^ | - | - |
| Parity | F_1,631_ = 0.92, p=0.337 | F_1,630_ = 1.25, p=0.264^a^ | - |
| SRLV serological status of a doe | F_5,607_ = 1.27, p=0.277 | F_5,608_ = 1.20, p=0.308 | F_5,610_=1.35, p=0.242 |

^a^ variables eliminated from MLM at subsequent steps
